# Short-term temporal memory in idiopathic and *Parkin*-associated Parkinson’s disease

**DOI:** 10.1038/s41598-018-25751-8

**Published:** 2018-05-16

**Authors:** Bertrand Degos, Ilhame Ameqrane, Sophie Rivaud-Péchoux, Pierre Pouget, Marcus Missal

**Affiliations:** 1 Neurology department, Parkinson’s disease expert centre, Salpêtriêre Hospital, AP-HP, Paris, France; 2 Sorbonne Universités, UPMC Univ Paris 06, Inserm U1127, CNRS UMR 7225, UM 75, ICM, F-75013 Paris, France; 3 Institute of Neuroscience (IONS), Cognition and Systems (COSY), Université catholique de Louvain, Avenue Mounier 53 bte B1.53.04 1200, Brussels, Belgium; 4 Neurology Unit, Avicenne University Hospital, AP-HP, Bobigny, France; 5 Present Address: Center for Interdisciplinary Research in Biology, Collège de France, UMR CNRS 7241/INSERM 1050, Labex Memolife, Paris, France

## Abstract

In a rapidly changing environment, we often know when to do something before we have to do it. This preparation in the temporal domain is based on a ‘perception’ of elapsed time and short-term memory of previous stimulation in a similar context. These functions could be perturbed in Parkinson’s disease. Therefore, we investigated their role in eye movement preparation in sporadic Parkinson’s disease and in a very infrequent variant affecting the *Parkin* gene. We used a simple oculomotor task where subjects had to orient to a visual target and movement latency was measured. We found that in spite of an increased average reaction time, the influence of elapsed time on movement preparation was similar in controls and the two groups of PD patients. However, short-term temporal memory of previous stimulation was severely affected in sporadic PD patients either ON or OFF dopaminergic therapy. We conclude that the two different contributions to temporal preparation could be dissociated. Moreover, a short-term temporal memory deficit might underlie temporal cognition deficits previously observed in PD.

## Introduction

Parkinson’s disease (PD) is a neurodegenerative disease characterized by motor (resting tremor, rigidity, bradykinesia and postural instability) and non-motor symptoms^1^. Particularly, cognitive decline is frequently observed early in the evolution of the pathology, causing rapid impairment of functions like memory^2–7^, attention^8^ and executive functions^9^. Cognitive decline has important consequences on the quality of life of patients and often precedes dementia^10^. It has been shown repeatedly that temporal cognition is also affected in PD^11–13^. Temporal cognition refers to a large set of functions among which the ability to estimate and reproduce durations in different sensory modalities. It is also essential before movement preparation^14,15^. Temporal preparation refers to this ability to plan a motor response in expectation of an upcoming trigger^16,17^. For instance, temporal preparation occurs while waiting for a traffic light to turn green. This predictive capacity is based on stored memories of previously experienced similar situations containing temporal cues. In an experimental environment, temporal preparation is studied using a warning stimulus before the presentation of the target of an upcoming movement^18^. The warning stimulus can be a sound or any stimulus allowing the subject to prepare to an upcoming imperative stimulus, e.g. a visual target or a response button. The period between the warning and the imperative stimuli is often referred to as the foreperiod (FP)^19,20^. When the duration of the FP is randomly selected from a uniform distribution with the same probability, the reaction time of the motor responses decreases with elapsed time. This ‘FP effect’ has been observed in all species where it has been investigated and in several different sensorimotor domains. In the oculomotor domain, if a target appears in the periphery of the visual field after a variable FP, the latency of saccadic eye movements progressively decrease with increasing FP duration^21^. This effect could be explained by a perception of an increased likelihood that the target will appear as time elapses during the FP^22,23^. However, the FP effect could also be attributed to the influence of the previous FP duration on current temporal preparation^24^. Indeed, the behavior of humans and animals in response to a sensory stimulation is often partly determined by the history of previous similar stimulation held in short-term memory, even if this influence goes unnoticed by the subject and is implicit.

The aim of the present study was to investigate temporal preparation and short-term memory in PD patients ON or OFF antiparkinsonian medication. We also compared idiopathic PD (referred to as ‘iPD’) with a genetic variant of the disease (referred to as ‘*Parkin’*). Indeed, there is a small population of PD patients that develops a form of the disease early in life due to mutations of the *Parkin* gene that causes autosomal recessive juvenile parkinsonism^25^. *Parkin* are thought to express mostly motor symptoms and less cognitive deficits^26^. To our knowledge, there is no study that addressed temporal preparation and memory in *Parkin* patients. Therefore, we compared these two variants of the disease in order to better estimate the influence of the heterogeneity of PD on temporal preparation and short-term memory.

## Methods

### Participants

Seventeen healthy control subjects (2 females, 15 males; 12% females) and twenty nine Parkinson’s disease (PD) patients took part to the study. Amongst the 29 PD patients, 19 presented the idiopathic form of the disease (referred to as ‘iPD’ patients, amongst which 3 females; 16% females) and 10 had a genetic variant of PD (referred to as ‘*Parkin’* patients; amongst which 2 females; 20% females). Although more females were present in the *Parkin* group of patients, there is no available experimental evidence or theoretical considerations suggesting that temporal preparation and short-term memory could be influenced by sex. *Parkin* patients were compound heterozygote/homozygote carriers of *Parkin* mutations. Sample size was determined by the availability of *Parkin* patients visiting the Neurology Department at the Salpêtrière Hospital in Paris, France. Ten patients could be recruited, an exceptionally large sample. Indeed, *Parkin* patients constitute only 10–20% of the population of early onset PD patients that itself constitutes only 15% of all PD patients^27^. Sample size was therefore intrinsically reduced for *Parkin* patients. The sample size of iPD patients was adapted to this limitation of sample size in *Parkin* patients. However, in each subjects, we collected approximately two hundred saccadic eye movements. All patients fulfilled the UKPDSBB criteria^28^. Subjects had normal or corrected to normal vision. All patients were taking medication to control their symptoms. All patients were taking levodopa and/or a dopaminergic agonist. The equivalent L-dopa daily dose was computed using the procedure described in Tomlinson *et al*.^29^. Patients of the present study were assessed using the Hoehn and Yahr scale^30^ and the motor part of the Unified Parkinson’s disease Rating Scale^31^ (UPDRS part III; see Table 1 for details). The Starkstein scale was used to measure apathy^32^. The Montreal Cognitive Assessment (MoCA) was used to assess dementia^33–35^. Subjects with a MoCA score of 25 or below were excluded.

**Table 1:** Demographics and clinical measures.

	Cont n = 17	iPD n = 19	*Parkin* n = 10	P-value t-test iPD/*Parkin*
Age (years)	57.4 ± 2.0	59.0 ± 1.7	42.6 ± 3.9	<0.001**
Age onset (years)	na	54.3 ± 1.9	23.3 ± 3.0	<0.001**
Duration (years)	na	4.6 ± 0.7	18.8 ± 2.8	<0.001**
Hoehn and Yahr (/5)	na	1.8 ± 0.1	1.6 ± 0.3	0.522
Starkstein ON (/42)	7.9 ± 0.8	11.4 ± 1.1	11.4 ± 2.1	0.992
Starkstein OFF (/42)	na	11.4 ± 1.3^#^	12.1 ± 2.4^#^	0.783
UPDRS III ON (/108)	na	13.6 ± 1.6	9.0 ± 1.7	0.081
UPDRS III OFF (/108)	na	22.3 ± 2.2	20.2 ± 3.4	0.603
MoCA (/30)	28.8 ± 0.2	28.3 ± 0.6	28.5 ± 0.5	0.844
L-Dopa equ (mg)	na	511 ± 56	443.5 ± 104.0	0.539

Cont: control subjects; iPD: idiopathic Parkinson’s disease; L-Dopa equ (mg): Levodopa equivalence in milligrams; na = not applicable. ^#^For the Starkstein score OFF, n = 14 in the iPD group and n = 8 in the *Parkin* group.

In order to test the influence of L-Dopa treatment, iPD patients were attributed to one of two groups. In the first group, patients were ON dopaminergic treatment during the first visit and testing (n = 9). Before the second visit, these patients withdrew from their current PD medications for the purpose of participating in this research project. The OFF state was practically defined as a 12-hour withdrawal of dopaminergic medication. In the second group of iPD patients (n = 10), the sequence of states was reversed: OFF during the first visit and then ON L-Dopa during the second visit. *Parkin* patients (n = 10) were always tested ON L-Dopa during the first visit and then OFF L-Dopa during the second visit. Given that *Parkin* patients are very infrequent and sometimes live at long distances, it was not possible to constitute a second group of these patients to test the OFF - ON transition. Amongst the 17 control subjects, 10 were tested twice as iPD patients, and 7 were tested only once. For all subjects, the two visits were separated by 7 to maximum 10 days and lasted approximately 1 hour each in all subjects.

### Ethical approval and informed consent

Experiments were approved by the local ethics committee (CPP Ile de France Paris VI, n° 212-A01056-37). Methods were carried out in accordance with relevant guidelines and regulations. Written informed consent was obtained from all participants. The study referred to as ‘PEDUPARK’ has been registered with clinicaltrials.gov (NCT02126475). All participants were informed about the purpose of the study and procedures before they gave their informed consent.

### Experimental paradigm

**Figure 1:**
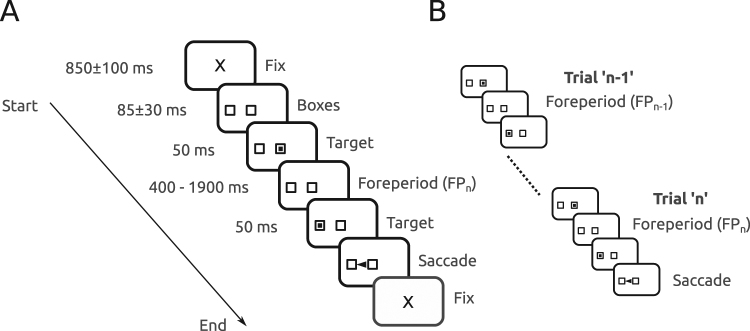
(**A**) Schematic description of the experimental paradigm. Subjects were facing a computer screen where the sequence of stimuli was presented. *Fix*: appearance of the fixation cross at the beginning of a trial. See Methods for details. (**B**) Schematic diagram illustrating the influence of short-term memory of the forepriod during the preceding trial (FP_n−1_) on saccadic latency during trial ‘n’ (FP_n_).

Subjects were facing a LCD screen which presented stimuli at a refresh rate of 140 Hz. An EyeLink 1000 infrared eye tracking system (SR Research, Mississauga, Ontario) was used to record eye movements at 1 KHz. Stimulus display and oculomotor data collection were synchronized on a frame-by-frame basis. Saccades were detected offline in MATLAB (MathWorks, Natic, MA) with a velocity threshold of 30 deg/sec. Figure 1A depicts the time line of stimuli presentation on the screen facing the subject. Each trial started with an initial fixation period of a small cross (0.7 deg) appearing on the screen for a random duration (850 ± 100 ms; see ‘X’ on Fig. 1A). At the end of this period, two empty square ‘boxes’ appeared on the screen (1.4 × 1.4 deg), one in the center of the screen and one 9 deg eccentric, randomly to the right or to the left. Afterwards, a square target was flashed in the central box for 50 ms. Extinction of the initial target indicated to subjects the beginning of the foreperiod (FP). Subjects were required to hold on fixation of the central box until another target was briefly presented for 50 ms in the eccentric box. The FP duration could take one of 4 different values with the same probability: 400 ms, 900 ms, 1400 ms and 1900 ms. Subjects were required to wait until stimulus appearance in the eccentric box to make a saccade (black arrowhead on Fig. 1A). Saccadic latency (reaction time) was defined as the time elapsed between the appearance of the eccentric target and movement onset. Figure 1B shows a schematic diagram to illustrate the influence of short-term memory of the forepriod during the preceding trial (FP_n−1_) on saccadic latency during trial’n’. Each sequence from the initial to the final fixation period will be referred to as a ‘trial’. Figure 1B shows two trials in succession. The foreperiod during the current trial (trial ‘n’) will be referred to as ‘FP_n_’. The foreperiod during the previous trial (trial ‘n−1’) will be referred to as ‘FP_n−1_’.

### Statistical analyses

The significance threshold α for all analysis was 0.01. All analyses were realized using analysis of variance (ANOVA) unless specified otherwise. Analyses were performed using SPSS 23 (SPSS Inc., Chicago, IL). Mixed-design ANOVA’s and multiple regression analysis were used. In all analyses, subject identity was used as a random factor to take into account the influence of uncontrolled variability observed between subjects. Results will be presented as mean ± standard error of the mean unless specified.

### Data availability

The datasets generated during and/or analysed during the current study are available from the corresponding author on reasonable request.

## Results

### Demographics and clinical characteristics

The mean age of iPD patients was 59.0 ± 1.7 years (n = 19) with a mean duration of the disease of 4.7 ± 0.7 years. The mean age of control subjects was 57.4 ± 1.7 years (n = 17). The mean age of *Parkin* patients was 43.0 ± 3.9 years (n = 10) with a mean duration of the disease of 18.8 ± 2.8 years. *Parkin* patients were statistically younger (F[1, 27] = 20.74; P < 0.001) and had a longer duration of the disease (F[1, 27] = 40.28; P < 0.001). However, Hoehn and Yahr stages were similar in both groups (F[1, 27] = 0.42; P = 0.52; NS) as well as the UPDRS III score ON L-Dopa treatment (F[1, 27] = 6.80; P = 0.015; NS; Table 1). The mean daily levodopa equivalent dose was similar in iPD (511 ± 56 mg, n = 19) and *Parkin* patients (444 ± 104 mg; F[1, 27] = 0.39; P = 0.54; NS; see Table 1). The mean age of iPD and control subjects was not statistically different (F[1, 34] = 5.03; P = 0.483; NS).

### Saccades in iPD and Parkin patients

**Figure 2:**
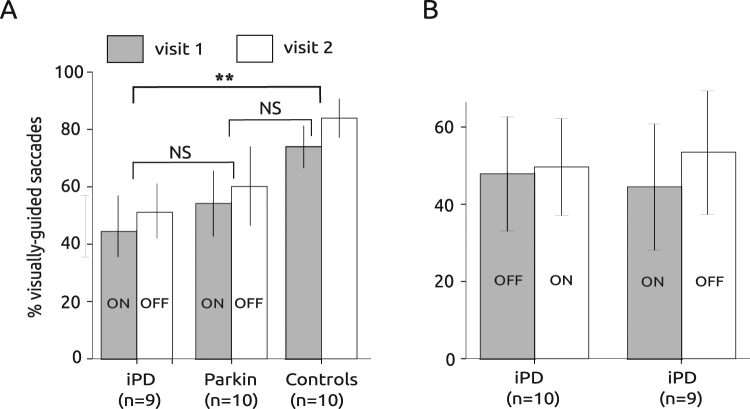
Saccadic hypokinesia in PD patients. (**A**) Percentage of visually-guided saccades towards the eccentric visual target in the different groups of subjects. Note that PD patients performed rather poorly compared with healthy controls. Data from subjects tested twice (visit 1 & visit 2). (**B**) Percentage of visually-guided saccades in the two groups of iPD patients. **Indicates a statistically significant differences (P < 0.01).

Given that an oculomotor form of hypokinesia could be observed in patients, we firstly compared the percentage of executed visually-guided saccades in patients and controls (see Fig. 2). At least two blocks of 100 trials were presented to all subjects (T_T_ = 200 trials total) and a total of 100 to 200 saccades (T_S_) were recorded per subject. The percentage of executed saccades was computed as [(T_S_/T_T)_*100]. To compare the percentage of saccades executed in the different groups, a repeated-measures ANOVA with subject group as between subjects factor and visit as within subject factor was performed. In this test, we used the ON-OFF group of iPD patients, in order to compare it with *Parkin* patients who were tested only with this same sequence of treatments (iPD ON-OFF group, n = 9; *Parkin*, n = 10; controls, n = 10; see Fig. 2). We found that the percentage of saccades significantly increased between visits (F[1] = 20.807; P < 0.001) and that subject group had a significant influence on the percentage of saccades (F[2] = 6.423; P = 0.005). However, there was no significant interaction between factors (F[2] = 0.018; P = 0.983). A post-hoc Bonferroni test suggests that on average the percentage of saccades was higher in controls than in iPD patients (P = 0.005) but not different between controls and *Parkin* patients (P = 0.073) or between iPD and *Parkin* patients (P = 0.757).

In iPD patients, we also compared the percentage of saccades between the two groups who experienced a different order of treatments (ON-OFF versus OFF-ON). In iPD patients, treatment order had no significant influence (F[1] = 0.001; P = 0.977). The percentage of visually-guided saccades did not significantly increase between visits (F[1] = 0.527; P = 0.473) and there was no significant interaction between factors (F[1, 3] = 0.234; P = 0.631; see Fig. 2B).

In summary, statistically less visually-guided saccades were observed in iPD patients than in controls. *Parkin* patients apparently made less-visually guided saccades than controls but this difference did not reach statistical significance. In iPD patients, dopaminergic treatment did not significantly alter the percentage of saccades observed that tended to increase with repetition (without reaching statistical significance).

### Temporal preparation in Parkinson’s disease

**Figure 3:**
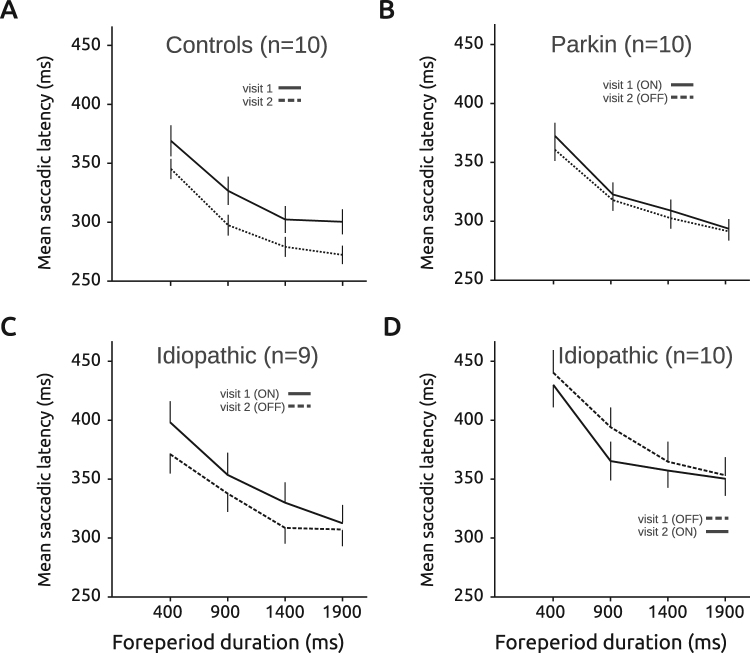
The foreperiod effect. Saccadic latency decreased as a function of increasing delay duration in controls (**A**), *Parkin* (**B**) and iPD patients (**C**, ON-OFF group; **D**, OFF-ON group). Although saccadic latency could be affected by treatment, the foreperiod effect itself (the decreasing saccadic latency with increasing foreperiod duration) was not affected by dopaminergic medication. Data from subjects tested twice (visit 1 & visit 2).

The foreperiod effect is a behavioral measure of temporal preparation that could be affected in PD. Figure 3 shows the relationship between saccadic latency and foreperiod (FP) duration in the 4 groups of subjects (controls, *Parkin*, iPD ON_1_ – OFF_2_ and OFF_1_ – ON_2_). Figure 3A shows that, in control subjects, there was a significant influence of FP duration on the latency of visually guided saccades (ANOVA with FP duration as fixed factor and subject as random factor; F[3, 27.363] = 27.663; p < 0.001; n_1_ = 1508 saccades; n_2_ = 1702 saccades; n = 10 subjects). As expected, saccadic latencies decreased with increasing FP duration (the FP effect). Although latencies tended to decrease on average between visit 1 and visit 2, this effect did not reach statistical significance (F[1, 9.037] = 7.304; p = 0.024; NS). Moreover, there was no significant interaction between FP duration and visit (F[3, 28.415] = 0.446; p = 0.722). In *Parkin* patients (see Fig. 3B), FP duration also had a significant effect on movement latency (F[3, 30.807] = 47.120; p < 0.001) but dopaminergic treatment had no effect (F[1, 9.242] = 0.550; p = 0.477; n_ON_ = 2272 saccades, n_OFF_ = 2544). No interaction between FP duration and treatment was found (F[3, 31.197] = 0.947; p = 0.430; n = 10 patients). In iPD patients, we tested the two different transitions between states in two different groups of subjects (ON_1_-OFF_2_ transition, see Fig. 3C; OFF_1_-ON_2_ transition, see Fig. 3D). In the ON_1_-OFF_2_ group (n = 9), FP duration (F[3, 29.950] = 9.382; p < 0.001; n_ON_ = 853 saccades, n_OFF_ = 1021) and treatment (F[1, 11.033] = 13.094; p = 0.004) had a significant effect on latencies but no significant interaction between FP duration and treatment was found (F[3, 30.982] = 1.056; p = 0.382). Saccadic latencies were longer during visit 1 ON L-Dopa but the FP effect remained unaltered. In the OFF_1_-ON_2_ group of iPD patients (n = 10), FP duration had a significant effect on latencies (F[3, 33.080] = 21.060; p < 0.001; n_OFF_ = 932 saccades, n_ON_ = 1021) but not dopaminergic treatment (F[1, 11.786] = 0.277; p = 0.608). Here also, no significant interaction between FP duration and treatment was found (F[3, 36.503] = 0.554; p = 0.649).

These results show that the FP effect was similar whether subjects were ON or OFF L-Dopa (absence of FP duration x dopaminergic treatment interaction). A significant influence of dopaminergic treatment on average saccadic latencies was present in the ON_1_-OFF_2_ iPD group, but there was no significant influence of the treatment on the FP effect itself. Therefore, we conclude that the FP effect did not depend on dopaminergic medication. Alteration of mean reaction time could be dissociated from the FP effect.

### Influence of the short-term temporal memory

**Figure 4:**
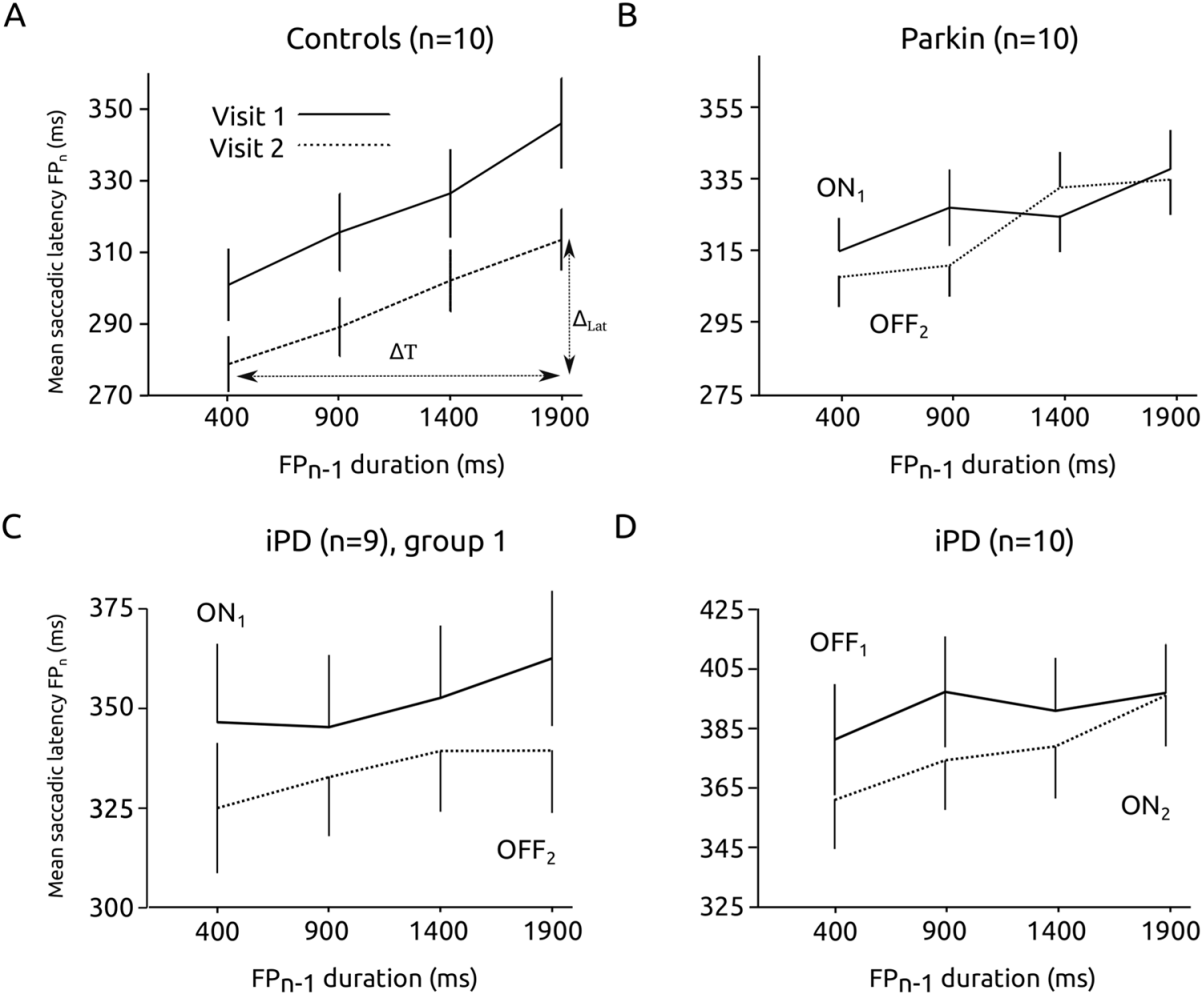
Influence of the duration of previous foreperiod (FP_n−1_) on saccadic latency during the current foreperiod (FP_n_) in the different groups of subjects. The influence of the previous foreperiod was significant only in controls (**A**) and *Parkin* patients (**B**) but not in iPD patients (**C**, ON_1_-OFF_2_ group; **D**, OFF_1_-ON_2_). See text for statistics.

It has been shown that the FP effect could depend heavily on the previously experienced FP duration^24^. This influence could be characterized as a sequence or temporal short-term memory effect. Indeed, the content of a short-term temporal storage (duration of FP_n−1_) could be influencing saccadic latency during the current foreperiod (FP_n_). Given that short-term memory is essential for executive functions, it could be a reliable indicator of cognitive decline in PD. Figure 4A shows the influence of FP_n−1_ duration on average saccadic latency during FP_n_ in controls. Saccadic latency tended to regularly increase with increasing FP_n−1_ duration during both visits, and a significant effect of FP_n−1_ on saccadic latency was found (mixed-design ANOVA with FP_n−1_ as fixed factor and subject as random factor: F[3, 28.344] = 28.199; P < 0.001; n = 3210 saccades total). However, there was no significant influence of visit (F[1, 9.037] = 8.403; P = 0.018) and no significant interaction between fixed factors (F[3, 28.195] = 0.873; P = 0.467). A similar trend was found in *Parkin* patients. Indeed, on average saccadic latency increased with increasing FP_n−1_ duration (F[3, 33.918] = 11.646; P < 0.001; n = 4816 saccades) but there was no significant influence of visit (F[1, 9.241] = 0.248; P = 0.630) and no significant interaction between fixed factors (F[3, 34.476] = 1.577; P = 0.213).

**Figure 5:**
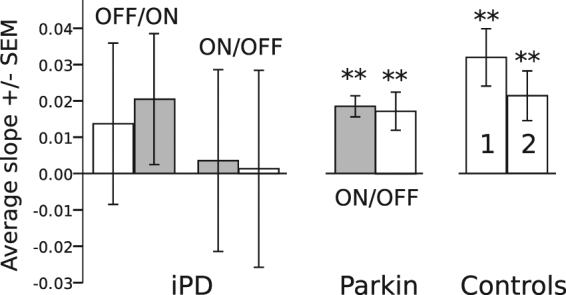
Short-term temporal memory deficit in PD patients. Average slopes in the different groups. Statistics: iPD: OFF_1_ (t = 0.12; df = 9; P = 0.25) – ON_2_ (t = 2.27; df = 9; P = 0.05); iPD: ON_1_ (t = 0.29; df = 9; P = 0.78) – OFF_2_ (t = 0.1; df = 8; P = 0.92); *Parkin*: ON_1_ (t = 12.79; df = 9; P < 0.001) – OFF_2_ (t = 6.58; df = 9; P < 0.001); controls: visit 1 (t = 8.10; df = 9; P < 0.001) – visit 2 (t = 6.24; df = 9; P < 0.001). Data from subjects tested twice (visit 1 & visit 2).

The influence of short-term temporal memory was statistically similar in controls and *Parkin* patients. However, the situation was different in iPD patients. Figure 4C,D show the relationship between FP_n−1_ duration and saccadic latency during FP_n_ in iPD patients (group 1: ON_1_ – OFF_2_, Fig. 4C; group 2: OFF_1_ – ON_2_, Fig. 4D). In group 1, saccadic latency did not increase with increasing FP_n−1_ duration (F[3, 33.049] = 1.180; P = 0.332; n = 1874 saccades). There was a significant influence of visit (F[1, 10.484] = 13.196; P = 0.004) but no significant interaction between fixed factors (F[3, 49.654] = 0.315; P = 0.815). These results suggest an absence of influence of short-term memory and a small test-retest effect on average saccadic latencies. In group 2, there was no significant effect of FP_n−1_ on saccadic latency (F[3, 44.759] = 3.041; P = 0.039; n = 1953 saccades), no significant influence of visit (F[1, 12.358] = 0.629; P = 0.443) and no significant interaction between fixed factors (F[3, 37.090] = 0.337; P = 0.798). To better capture this effect, we computed for each subject the average slope of the relationship between saccadic latency during trial’n’ and FP_n−1_ duration (see Fig. 4A): Slope = Δ_LAT_/ΔT with: Δ_LAT_ = [(average latency of saccades for FP_n−1_ = 1900 ms)-(average latency of saccades for FP_n−1_ = 400 ms)]; ΔT = 1900–400 = 1500 ms. Figure 5 shows the average value of the slope for each group. If there were a modulation of saccadic latency with short-term temporal memory, the average value of the slope should be different from zero. In iPD patients, the slope was more variable than in *Parkin* patients or controls. We applied a one sample two-tailed t-test with a test-value of zero for each group. In iPD patients, there was no significant difference between average slope values and zero in either the OFF_1_ – ON_2_ or the ON_1_ – OFF _2_ group (P > 0.01). However, slopes were significantly different from zero in *Parkin* patients and controls (see legend of Fig. 5 for detailed statistics). We found no correlation between the average slope values for each subject and demographics (age, duration of the disease), L-Dopa equivalent or UPDRS III score (Pearson correlation, two-tailed test, P > 0.01).

**Figure 6:**
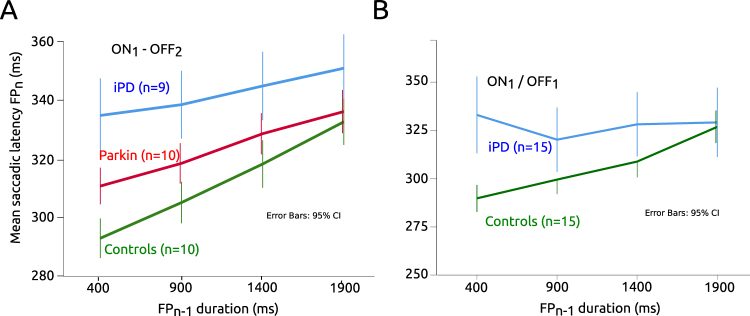
(**A**) Between group comparison of the relationship between previous FP duration and saccadic latency. (**B**) Same analysis on an additional data set with controls and iPD patients differing by <1 year of age.

In order to determine whether on average there was a different linear relationship between FP_n−1_ and saccadic latency between iPD and Parkin patients with respect to controls, a Mixed-model multiple linear regression analysis was performed with subjects as random factor, group as fixed factor and FP_n−1_ as co-variate. This analysis should give a significant interaction term (moderator effect) if there is a different linear relationship between variables in iPD and *Parkin* patients with respect to controls. Figure 6A shows the relationships between FP_n−1_ and saccadic latency for the 2 groups of patients tested ON_1_ – OFF _2_ (*Parkin* and iPD) and controls. It can be seen that saccadic latency increased rapidly with FP_n−1_ duration in controls but that this relationship could have a shallower slope in patients. For *Parkin* patients the slope of the relationship was apparently intermediate between the other two groups. If there is a moderator effect in the mixed-model multiple linear regression analysis, then it could be hypothesized that the influence of short-term temporal memory on saccadic latency is different between groups of subjects taking controls as reference. We did not find a significant main effect of patient group (F[2, 9894] = 1.836; P < 0.160) but a significant main effect of FP_n−1_ duration (F[1, 24.804] = 82.359; P < 0.001). However, we found that the moderator effect was not significant (F[2, 22.207] = 2.286; P = 0.125). Therefore, based on this data set, we could not conclude that there is a different linear relationship between FP_n−1_ duration and saccadic latency between groups. This conclusion could be due to the small sample of subjects. In order to lift this limitation, we pooled iPD subjects of the ON_1_-OFF_2_ and OFF_1_-ON_2_ groups together. This pooling is legitimated by the observation that dopaminergic treatment had no significant influence on short-term memory in iPD patients (see above). iPD patients were either ON_1_ or OFF_1_. This could not be done for *Parkin* patients. Indeed, there is not enough *Parkin* patients in France to constitute two groups. Moreover, given that age is a major factor of general decline that could occur rapidly, we collected additional data on 7 male healthy subjects in order to create cohorts of equal sizes that have less than a year of age difference between controls and iPD patients (controls: 57.9+/−2.0 years, n = 15; iPD patients: 57.5+/−1.9 years, n = 15; ~3000 saccades in each group). These additional control subjects were tested only once (visit 1 only). We found no significant difference in age between groups (F[1] = 0.022; P = 0.883). Here also, the average slopes of the FP_n−1_/saccade latency relationship were significantly different from zero in controls (t[14] = 4.825; P < 0.001) but not in iPD patients (t[14] = 0.280; P = 0.783). Moreover, we carried out a similar Mixed-model multiple regression analysis with these two groups of subjects. In this case, we found that FP_n−1_ duration (F[1, 28.040] = 10.052; P = 0.004) had a significant effect but not subject group (controls or iPD; F[1, 3347] = 3.732; P = 0.053). However, a significant interaction (moderator effect) was found between independent variables (F[1, 28.040] = 8.447; P = 0.007; see Fig. 6B). This result suggests that the linear FP_n−1_/saccade latency relationship was indeed different between iPD patients and controls.

## Discussion

We found that in all subjects and patients groups, temporal preparation (the foreperiod effect) was preserved. Given the motor deficits observed in PD, several authors have tested the FP effect in these patients^36–38^. All these previous studies confirmed that the FP effect was observed (but see [38] for a dissociation between reflexive and voluntary behaviors). Accordingly, in the present study, in both groups of PD patients, latencies steadily decreased as time elapsed during the delay period. Observation of a preserved variable FP effect in PD patients is not surprising. Indeed, this effect is very robust in several neurological diseases, and does not depend on an explicit conscious manipulation of temporal information that is affected in PD^12^. Stated differently, the implicit monitoring of elapsed time is functional in PD patients. Given that the duration of the previous FP (FP_n−1_) has been shown to influence movement latency and the FP effect in healthy subjects, we analyzed the influence of FP_n−1_ on saccadic latency. Within-group analyses revealed that memory of the previous FP duration affected movement latency during the current trial’n’ in controls and *Parkin* patients but not in iPD patients. The slope analysis confirmed this observation. A Mixed-model multiple linear regression analysis revealed that the posited linear relationship between FP_n−1_ and saccadic latency was not statistically different between groups. This absence of effect might be related to the small sample size dictated by the small population of *Parkin* patients available in France. However, comparison between controls and iPD subjects matched for a difference of age of less than a year and independently of dopaminergic status showed that memory of previous FP played a significantly different role btween these two groups. Most cognitive processes rely on working memory that could be defined as the ability to maintain and manipulate meaningful information during a short time period for usage in complex tasks like language, learning and reasoning^39^. Several studies suggest an impairment of working memory in PD patients^40^. Indeed, the pathophysiological process of PD might affect working memory. Short-term memory could be described as the input buffer to working memory. If working memory is not properly fed with temporal information, then all time-dependent cognitive abilities might be affected. The ability to keep track of the history of previous foreperiods (FP_n−1_) is essential in order to build an internal representation of the likelihood of future foreperiods (FP_n_, FP_n+1_, etc…). Memory is useful if it helps to anticipate or predict future events. If prediction based on short-term memory does not operate properly, iPD patients might be in a constant state of unpreparedness when the target appears, generating longer latency responses. It could be argued that a loss of cognitive flexibility could explain observed effects. Cognitive flexibility could be defined as the capacity to weight different alternatives when faced with a complex problem. It involves also the capacity to shift attention from one rule valid in a given context to a new rule. Idiopathic PD patients tend to perseverate if rules to execute a given task are unexpectedly changed, showing an obvious lack of cognitive flexibility^41–46^. Therefore, one could argue that reduced cognitive flexibility is sufficient to explain the perturbed sequence effect or reduced short-term memory observed in iPD patients in the present study. For at least two reasons we believe this hypothesis could be rejected. Firstly, it should be emphasized that cognitive flexibility is always tested in tasks where information must be explicitly manipulated in working memory in order to achieve a particular goal. This is not the case here. Indeed, no instruction about trial sequence was required from subjects. They were asked to ‘make a saccade to the eccentric target when it appears in the periphery’. Secondly, in the present experimental paradigm, if loss of cognitive flexibility were a valid explanation, it should manifest itself by perseverance based on previous information and therefore a *stronger* influence of FP_n−1_. Another alternative explanation to reduced short-term temporal memory is that bradyphrenia or bradykinesia could explain observed effects. Bradyphrenia is a general slowing of cognitive processes^1^ and bradykinesia is a slowing down of movements generally observed in iPD^47^. Could these two commonly observed phenomena in iPD explain observation of the present study? Firstly, a strong influence of the current FP (the foreperiod effect) was found in our patients in spite of an increased average saccadic latency (oculomotor hypo/bradykinesia). Therefore, implicit temporal processing during the *current* trial was not affected. However, this information was not subsequently used in the planning of the next saccadic eye movement. Secondly, cognitive abilities evaluated by the MoCA test did not differ between controls and iPD patients. Therefore, we hypothesize that a reduced or more variable influence of FP_n−1_ as presented here results of an impairment of temporal short-term memory (or sequence effect) and not of a general slowing of motor and cognitive processes that should be detected with the MoCA test.

In conclusion, the oculomotor variant of the variable foreperiod paradigm used in the present study confirmed that temporal preparation could depend of both an implicit ‘perception’ of elapsed time and short-term memory. The influence of elapsed time during the current foreperiod was not affected by PD or dopaminergic status even if saccadic latency tended to be longer in PD patients. A within-group analysis showed that the influence of the previous foreperiod (FP_n−1_) was present in controls and *Parkin* patients, but not in iPD patients. In iPD patients, a robust foreperiod effect could be observed in the absence of a short-term memory effect. The two different contribution to the foreperiod effect could be dissociated. However, we could not determine if there was a significantly different linear relationship between FP_n−1_ and saccadic latency in iPD and *Parkin* patients with respect to controls.
